# *APOC3* rs2070667 Associates with Serum Triglyceride Profile and Hepatic Inflammation in Nonalcoholic Fatty Liver Disease

**DOI:** 10.1155/2020/8869674

**Published:** 2020-11-26

**Authors:** Qing-Yang Xu, Han Li, Hai-Xia Cao, Qin Pan, Jian-Gao Fan

**Affiliations:** ^1^Department of Gastroenterology, Xinhua Hospital, School of Medicine, Shanghai Jiaotong University, Shanghai 200092, China; ^2^Department of Pediatric Gastroenterology, Xinhua Hospital, Shanghai Jiaotong University School of Medicine, Shanghai 200092, China; ^3^Shanghai Key Laboratory of Children's Digestion and Nutrition, Shanghai 200092, China

## Abstract

Single-nucleotide polymorphisms (SNPs) of apolipoprotein C3 (*APOC3*) play important role in lipid metabolism, and dyslipidemia underlies nonalcoholic fatty liver disease (NAFLD). But the correlation of serum lipidomics, *APOC3* SNPs, and NAFLD remains limited understood. Enrolling thirty-four biopsy-proven NAFLD patients from Tianjin, Shanghai, Fujian, we investigated their *APOC3* genotype and serum lipid profile by DNA sequencing and ultraperformance liquid chromatography-tandem mass spectrometry (UPLC-MS/MS), respectively. Scoring of hepatocyte steatosis, ballooning, lobular inflammation, and liver fibrosis was then performed to reveal the role of lipidomics-affecting *APOC3* SNPs in NAFLD-specific pathological alterations. Here, we reported that *APOC3* SNPs (rs4225, rs4520, rs5128, rs2070666, and rs2070667) intimately correlated to serum lipidomics in NAFLD patients. A allele instead of G allele at rs2070667, which dominated the SNPs underlying lipidomic alteration, exhibited downregulatory effect on triacylglycerols (TGs: TG 54 : 7, TG 54 : 8, and TG 56 : 9) containing polyunsaturated fatty acid (PUFA). Moreover, subjects with low-level PUFA-containing TGs were predisposed to high-grade lobular inflammation (TG 54 : 7, rho = −0.454 and *P* = 0.007; TG 54 : 8, rho = −0.411 and *P* =0.016; TG 56 : 9, rho = −0.481 and *P* = 0.004). The significant correlation of *APOC3* rs2070667 and inflammation grading [G/G vs. G/A+A/A: 0.00 (0.00 and 1.00) vs. 1.50 (0.75 and 2.00), *P* = 0.022] further confirmed its pathological action on the basis of lipidomics-impacting activity. These findings suggest an inhibitory effect of A allele at *APOC3* rs2070667 on serum levels of PUFA-containing TGs, which are associated with high-grade lobular inflammation in NAFLD patients.

## 1. Introduction

Nonalcoholic fatty liver disease (NAFLD) reflects an important pathological syndrome demonstrating hepatocyte steatosis, ballooning, and lobular inflammation, with clinical outcomes of liver fibrosis/cirrhosis, hepatocellular carcinoma, and related mortality [1–8]. By its rapid growing incidence in the recent decades, NAFLD has already been a leading cause of chronic liver diseases worldwide [9–13]. Aberrant lipid metabolism, which facilitates the hepatocyte steatosis, oxidative stress, hepatic injury, and inflammation [12, 14–16], is now widely accepted to serve as a fundamental etiology that results in the NAFLD-inducing “multiple hits” [10, 17, 18].

Nowadays, multiple studies reveal an intimate association of single-nucleotide polymorphisms (SNPs) in the lipometabolism-related genes [19–24] and the genetic susceptibility of NAFLD [25–32]. Among these genes, *APOC3* encodes apolipoprotein C3 (apoC3), which is the critical inhibitor of lipoprotein lipase (LPL) in chylomicron (CM) remnants and very-low-density lipoprotein (VLDL) [33–35]. The modulatory effect of apoC3 on LDL receptor (LDLR) activation causes the reduction of uptake and integration of triglyceride- (TG-) rich particles in hepatocytes [33–35]. Loss-of-function mutations of *APOC3* (R19X, IVS2+1G→A, IVS3+1G→T, A43T, and V50M) [33, 36] have been well described to correlate to plasma triglycerides [37] and related cardiovascular diseases (coronary heart disease, ischemic cardiovascular disease, etc.) in heterozygous carriers [33, 36]. The critical role of *APOC3* in lipid metabolism, together with the dyslipidemia caused by *APOC3* mutations [38, 39] and apolipoprotein C3 deficiency [33, 36], suggests an association of *APOC3* SNPs and dyslipidemia-based NAFLD. However, this *APOC3* SNPs and NAFLD association remains limited, yet controversial, understood [32, 40–43]. In brief, *APOC3* variants (C-482T or T-455C) in the promoter are reported to predispose subjects to NAFLD by Petersen et al. [32], but this observation was disproved by Kozlitina et al. [41]. Thus, the correlation of *APOC3* SNPs, serum lipids, and NAFLD needs further exploration.

We, therefore, performed *APOC3* sequencing, serum lipidomic detection, and pathological evaluation in Chinese Han patients with biopsy-proven NAFLD. The association among *APOC3* SNPs, serum lipid profile, and pathological scoring was then investigated to uncover the lipidomics-based intervention of *APOC3* polymorphisms on NAFLD.

## 2. Materials and Methods

### 2.1. Study Subjects

Thirty-four (male : female = 19 : 15) Chinese Han patients with biopsy-proven NAFLD were recruited from Xinhua Hospital, Shanghai (*n* = 17); Tianjin Hospital of Infectious Diseases, Tianjin (*n* = 9); and Zhengxing Hospital, Zhangzhou, Fujian (*n* = 8) during January 2012 and June 2013 in this cross-sectional study (Table 1). The following criteria were employed for patient exclusion: (1) drinking history or excessive alcohol consumption, (2) virus hepatitis, (3) steatosis-related chronic liver diseases, and (4) liver transplantation [44–47]. Each participator provides 1 mL total blood for further detection. Institutional approval of this study was obtained from Xinhua Hospital Research Ethics Committee, and informed consent was issued by each participant. All methods in this study were conducted in accordance with the approved guidelines and the Declaration of Helsinki.

### 2.2. DNA Isolation and Genotyping of *APOC3* SNPs

After centrifugation of 500 *μ*l total blood, DNA was extracted from peripheral mononuclear cells by QiAamp DNA Mini Kit (Qiagen, Venlo, Netherlands). The concentration and quality of DNA were verified using NanoDrop® ND-1000 (Thermo Fisher Scientific, Waltham, MA, USA) and 0.8% agarose gel electrophoresis. Thereafter, primers of five *APOC3* SNPs (rs4225, rs4520, rs5128, rs2070666, and rs2070667) were designed on the basis of dbSNP database (https://www.ncbi.nlm.nih.gov/snp/) to construct a custom Ion AmpliSeq panel (Thermo Fisher Scientific, Waltham, MA, USA). The emulation polymerase chain reaction (PCR) of the template DNA was processed using the Ion OneTouch 2 System (Thermo Fisher Scientific, Waltham, MA, USA) according to the manufacturer's instructions. *APOC3* SNPs were successively genotyped according to the following procedures: (1) DNA sequencing by Ion 318 Chip on the Ion PGM™ System (Thermo Fisher Scientific, Waltham, MA, USA) and (2) data analysis by the Auto-user software (Life Technology, Gaithersburg, MD, USA) [48].

### 2.3. Lipidomics Analysis

Serum sample (40 *μ*l) from each NAFLD patient was subjected to serum lipidomic analysis using Triple TOF 5600 mass spectrometer (AB SCIEX, Framingham, MA, USA) by means of untargeted ultraperformance liquid chromatography-tandem mass spectrometry (UPLC-MS/MS). Firstly, a Waters BEH C8 column (2.1 mm × 100 mm and 1.7 *μ*m) was used for lipid separation. The mobile phases were composed of 3 : 2 (v/v) ACN/H_2_O (10 mM AcAm, phase A) and 9 : 1 (v/v) IPA/ACN (10 mM AcAm, phase B). A 20-minute elution gradient program was run at the flow rate of 0.26 mL/min and the column temperature of 55°C. The elution gradient stared with 32% B for 1.5 min and rose up linearly to 85% B at 14 min, then reached 97% B at 15.5 min for 2.5 min. It returned to 32% B within 0.1 min and held for 1.9 min for column equilibration.

The UPLC-MS/MS parameters conducted for lipid detection were summarized as follows: temperature of interface heater, 600°C in electrospray ionization (ESI) (–) and 500°C in ESI (+); ion spray voltage of MS: 4500 V in ESI (–) and 5500 V ESI (+); declustering potential: 100 V ESI (–) and 100 V ESI (+); collision energy: 10 V ESI (–) and 10 V ESI (+). Thirteen quality control (QC) samples were randomly inserted into the testing sequence.

Raw data obtained was identified using LipidView/PeakView (AB SCIEX, Framingham, MA, USA) and quantified using MultiQuant 2.0 (AB SCIEX, Framingham, MA, USA). The relative standard deviation of 239 serum lipids in QC samples was measured against the internal standards [49].

### 2.4. Hepatic Histopathologic Assessment

Liver specimens from each patient were obtained by ultrasound-guided needle biopsy. Each sample was then treated by 10% formalin fixing, paraffin embedding, slicing, and hematoxylin & eosin (HE) and Masson's trichrome staining in succession. Three pathologists who were not aware of the study assessed the NAFLD-related pathological characteristics according to the steatosis, activity, and fibrosis (SAF) scoring method as follows: (1) steatosis (S0, <5%; S1, 5-33%; S2, 34-66%; S3, >66%); (2) activity: sum of lobular inflammation (0, no foci per 200× field; 1, <2 foci per 200× field; 2, 2-4 foci per 200× field; 3, >4 foci per 200× field) and ballooning (0, none; 1, few balloon cells; 2, many cells/prominent ballooning); (3) fibrosis (F0, none; F1, perisinusoidal or portal fibrosis; F2, perisinusoidal and periportal fibrosis without bridging; F3, bridging fibrosis; F4, cirrhosis) [1, 50–52].

### 2.5. Statistical Analysis

All data were expressed as means ± standard deviation (SD). Unpaired Student's independent *t*-test was used to investigate the differences in serum lipidomics after examining the normality of data (*P* ≥ 0.1) by Kolmogorov-Smirnov test. Spearman's correlation was performed to evaluate the association of serum lipid profile and hepatic histological parameters. Differences in histological parameters were analyzed by Mann-Whitney *U* test. Analyses were performed using SPSS21.0 (SPSS Inc., Chicago, IL, USA) with a two-side significant criterion at *P* < 0.05.

## 3. Results

### 3.1. *APOC3* SNPs Correlated with Serum Lipidomics in NAFLD Patients

Detecting *APOC3* SNPs and 239 serum lipids, the effect of *APOC3* polymorphisms on serum lipidomics was subjected to evaluation. Dramatically, there was a distinct correlation between *APOC3* SNPs (rs4225, rs4520, rs5128, rs2070666, and rs2070667) and serum lipidomics in our NAFLD cohort (Table 2). *APOC3* SNP-related lipids, including ceramide (Cer), diacylglycerol (DG), choline plasmalogen (PCO), phosphatidylethanolamine (PE), ethanolamine plasmalogen (PEO), phosphatidylinositol (PI), and triacylglycerol (TG), characterized the lipidomic alteration (Table 2).

### 3.2. G/A or A/A Genotype of *APOC3* rs2070667 Exhibited Downregulatory Effect on Serum Lipid Profile

In contrast to most *APOC3* SNPs exhibiting limited-scale lipidomic association, *APOC3* rs2070667 exerted wide-range impacts on serum lipids of Cer, DG, PCO, and TG with statistical significance (Table 2). When compared to those with G/G genotype, NAFLD patients carrying G/A+A/A genotypes at *APOC3* rs2070667 demonstrated statistically lowered levels of TGs (TG 54 : 7, TG 54 : 8, and TG 54 : 9), whereas obvious higher levels of Cer (Cer 42 : 1; 2), DG (DG 36 : 4), and PCO (PCO 38 : 4 and PCO 40 : 4) (Table 2).

### 3.3. *APOC3* rs2070667-Dependent Low-Level TGs Associated with High-Grade Lobular Inflammation

**Figure 1:**
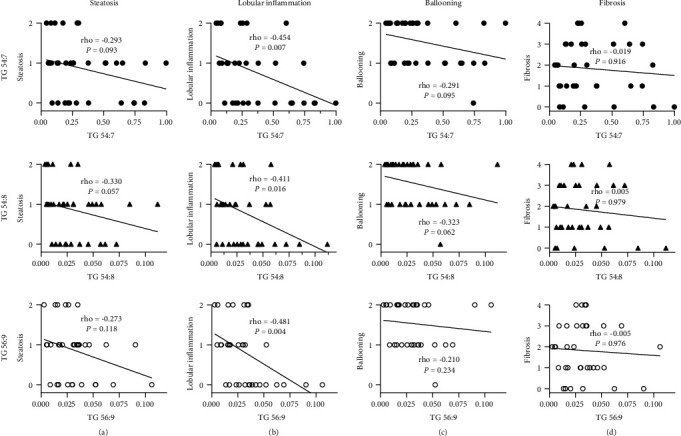
Lower-level TGs were correlated with higher-grade lobular inflammation in nonalcoholic fatty liver disease patients. Scatters reflected the serum levels of TGs (TG 54 : 7, TG 54 : 8, and TG 56 : 9) and pathologic scoring (steatosis, lobular inflammation, ballooning, and fibrosis) in patients with nonalcoholic fatty liver disease. Linear fit lines were presented with rho corresponding to the correlation of TGs and pathological characteristics. Inverse correlation between TG 54 : 7, TG 54 : 8, and TG 56 : 9 and lobular inflammation was observed with statistical significance. The rho values of fit lines in (b) were similar.

To shed light on the interaction between serum lipidomics and NAFLD, we further investigated the association of *APOC3* rs2070667-related differential serum lipids and NAFLD-specific pathological disorders (hepatocyte steatosis, lobular inflammation, ballooning, and liver fibrosis). Interestingly, polyunsaturated fatty acid- (PUFA-) containing TGs among these ones showed a negative correlation with lobular inflammation in similar Spearman coefficients (TG 54 : 7, rho = −0.454 and *P* = 0.007; TG 54 : 8, rho = −0.411 and *P* =0.016; TG 56 : 9, rho = −0.481 and *P* =0.004) (Figure 1(b)). Being compared to those carrying G/G at *APOC3* rs2070667, the NAFLD patients with G/A or A/A genotype were characterized by low serum levels of PUFA-containing TGs and high-grade lobular inflammation (Figure 1(b)). Other pathological characteristics of NAFLD, especially hepatocyte steatosis, shared the mild correlation of serum PUFA-containing TGs in an inverse manner (Figures 1(a), 1(c), and 1(d)).

### 3.4. NAFLD Patients Carrying A Allele at *APOC3* rs2070667 Showed Severe Lobular Inflammation

**Figure 2:**
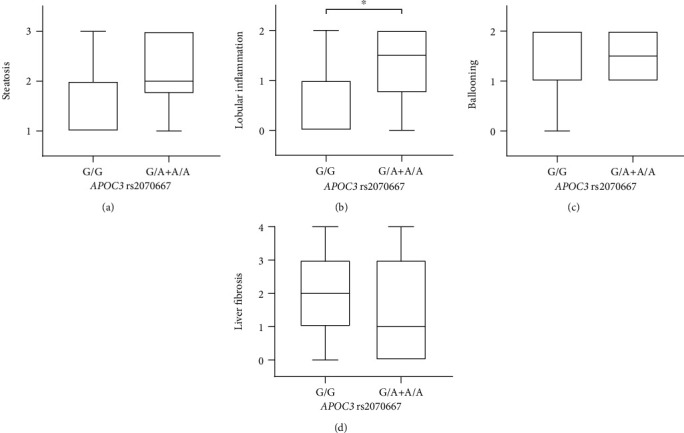
Nonalcoholic fatty liver disease patients carrying G/G vs. G/A+A/A genotype at *APOC3* rs2070667 demonstrated higher-grade lobular inflammation. Box plots indicated the differences in pathological characteristics of steatosis, lobular inflammation, ballooning, and fibrosis between nonalcoholic fatty liver disease patients with G/G vs. G/A+A/A genotype at *APOC3* rs2070667. An aggravation of lobular inflammation was documented in the NAFLD patients carrying A allele (G/A and A/A) at *APOC3* rs2070667 in comparison to those with G allele. Mild upregulated steatosis was also presented in the patients carrying A allele. Results were presented as medians and interquartile range. ^∗^*P* < 0.05.

By scoring hepatocyte steatosis, lobular inflammation, ballooning, and liver fibrosis, we assessed the role of *APOC3* rs2070667 in NAFLD-related pathological characteristics. An aggravation of lobular inflammation and, to less extent, steatosis was documented in the NAFLD patients carrying A allele (G/A and A/A) at *APOC3* rs2070667 in comparison to those with G allele (lobular inflammation: G/G vs. G/A+A/A: 0.00 (0.00 and 1.00) vs. 1.50 (0.75 and 2.00), *P* = 0.022; hepatocyte steatosis: G/G vs. G/A+A/A: 2.00 (1.00 and 2.00) vs. 2.00 (1.75 and 3.00), *P* = 0.076) (Figures 2(a) and 2(b)). However, different alleles at *APOC3* rs2070667 showed insignificant effect on ballooning (G/G vs. G/A+A/A: 2.00 (1.00 and 2.00) vs. 1.50 (1.00 and 2.00), *P* = 0.744) and liver fibrosis (G/G vs. G/A+A/A: 2.00 (1.00 and 3.00) vs. 1.00 (0.00 and 3.00), *P* = 0.201) (Figures 2(c) and 2(d)). Thus, A allele at *APOC3* rs2070667 is suggested to underlie severe lobular inflammation in the NAFLD patients with close association of lowered levels of PUFA-containing TGs.

## 4. Discussion

Multiple researches have shed light on the versatile actions of *APOC3* in lipid metabolism [35, 38, 39, 53–56]. apoC3 within CM is capable of inhibiting the LPL-dependent CM-TG hydrolysis and the hepatic CM intake on a basis of apoE and LDL receptor-related protein (LRP) combination [35, 53]. Upregulated apoC3 in VLDL also retards its clearance [38, 39, 54–56]. In addition, plasma level of apoC3 exhibited a correlation to HDL lipids of cholesterol ester (CE), TG, free cholesterol (FC), phosphatidylcholine (PC), PCO, sphingomyelin (SM), DG, Cer, and LPC [37]. This lipometabolic activity of apoC3 suggests an association of *APOC3* SNPs and lipid metabolic traits [20, 23, 35, 37–39, 53–56].

Indeed, subjects with *APOC3* -2854 G/T demonstrate higher serum TG [57], whereas -482C>T in *APOC3* is related to the serum levels of TG and other lipids in the Chinese population [57]. An important role of polymorphisms tagging *APOC3* is further convinced in the occurrence of dyslipidemia by Genome-Wide Association Studies (GWAS) [58–60]. Integrating DNA sequencing and UPLC-MS/MS analysis, our study presented novel findings that a group of *APOC3* SNPs (rs4225, rs4520, rs5128, rs2070666, and rs2070667) exerted global impact on serum lipidomics in NAFLD patients. Moreover, *APOC3* rs2070667 among these ones was statistically associated with 7 differential serum lipids, including Cer, DG, PCO, and TGs.

Nowadays, there are growing evidences highlighting a crucial effect of serum lipids on NAFLD [61–64]. Relying on the lipidomics-based evolutionary algorithm, serum lipids of TG (16 : 0, 18 : 0, and 18 : 1), PC (18 : 1 and 22 : 6), and PCO (24 : 1 and 20 : 4) are put forward to be predictive biomarkers of NAFLD [61]. On the contrary, both serum LPCs and PUFA-containing phospholipids associate with the liver fat content in an inverse manner [61]. Decrease in serum palmitoyl-, stearoyl-, and oleoyl-LPC characterizes the mice with experimental nonalcoholic steatohepatitis (NASH) [62]. Nevertheless, FFA released from circulating TG and adipose tissue has been reported to contribute to hepatocellular FFA accumulation and steatosis [63, 64]. *APOC3* SNPs, therefore, are proposed to interfere in the NAFLD by lipidomic modulation.

When compared to those with G/G genotype, we documented significantly lowered levels of TGs (TG 54 : 7, TG 54 : 8, and TG 54 : 9) in NAFLD patients with G/A or A/A genotype at *APOC3* rs2070667. Moreover, there was a negative correlation between these PUFA-containing TGs and NAFLD-specific pathological characteristics, including lobular inflammation and steatosis, in comparable Spearman coefficients. In contrast to saturated fatty acids that upregulate the levels of proinflammatory cytokines, n-3 PUFAs have been described to attenuate the inflammation activity of liver by the reduction of proinflammatory cytokine (e.g., TNF-*α*, IL-1*β*, and IL-6) secretion, as well as the increase of anti-inflammatory cytokine (e.g., adiponectin) [65–67]. Their incorporation into the phospholipids of inflammatory cells may underlie these pharmacological actions, resulting in the improved membrane fluidity and modified lipid derivatives [65]. On the other hand, PUFAs prevent the liver from steatosis on a basis of SREBP-1c and lipogenic gene (e.g., FAS, ACC, and SCD-1) downregulation [68] and then alleviate the hepatic inflammation by an amelioration of oxidative stress [69, 70].

In the present study, we verified much higher SAF grade of lobular inflammation in NAFLD patients carrying A allele at *APOC3* rs2070667 in comparison to those with G allele. By the mild increase in their steatosis scoring, an association was highlighted between *APOC3* rs2070667 and hepatocyte steatosis. In result, *APOC3* rs2070667 is indicated to be responsible for the deteriorated pathological characteristics in NAFLD patients by, to a large extent, its inhibitory impact on the serum levels of PUFA-containing TGs. Contrastively, there are some literatures deny the role of *APOC3* SNPs in NAFLD [40, 71]. This disagreement may be partially attributed to the test of blood lipids in routine method, which is insufficient to distinguish numerous components and their alterations in serum lipidomics.

Being contrast to most filtered SNPs locating in the exons of *APOC3*, rs2070667 is found to be an intron-resided SNP with probably intact structure and catalytic activity of apoC3. Its effects on serum lipidomics and NAFLD could be attributed to the epigenetic regulations, such as DNA methylation and miRNA-based expressive tuning [72–75]. Furthermore, transcriptional regulation reflects another potential mechanism of its lipidomic and pathological roles because approximately 40% of transcription factor (TF) binding sites have been identified in the introns [76].

However, there are some limitations in the present study. First, lacking of quantitative apoC3 test remains a shortcoming in highlighting the mechanisms underlying effects of *APOC3* SNPs. In addition, untargeted UPLC-MS/MS instead of targeted UPLC-MS/MS was employed to investigate the effect of *APOC3* on serum lipidomics. Thus, number of unsaturated bonds, but not their location, in serum lipids could be identified in our experiments.

## 5. Conclusion

*APOC3* SNPs exhibit impact on the serum lipidomics of NAFLD patients. A allele at *APOC3* rs2070667 demonstrates predominantly downregulatory effect on the serum lipid profile. Low-level PUFA-containing TGs (54 : 7, 54 : 8, and 56 : 9) among these differential lipids display significant association with high-grade lobular inflammation. Therefore, NAFLD patients carrying A instead of G allele at *APOC3* rs2070667 may susceptible to hepatic inflammation upon the rs2070667-based alteration of serum TGs.

## Figures and Tables

**Table 1:** Basic information of population.

	Mean	SD
Age (years)	41.03	14.81
Weight (kg)	75.34	9.49
Waist (cm)	90.59	6.61
BMI (kg/m2)	26.90	3.13
ALB (mg/dl)	41.36	7.55
ALT (U/L)	70.47	56.19
AST (U/L)	52.04	34.78
TC (mg/dl)	4.70	0.64
TG (mg/dl)	1.71	0.70
HDL (mg/dl)	1.16	0.28
LDL (mg/dl)	2.70	0.56

SD: standard deviation; ALB: albumin; ALT: alanine aminotransferase; AST: aspartate aminotransferase; TC: total cholesterol; TG: total triglyceride; HDL: high-density lipoprotein; LDL: low-density lipoprotein.

**Table 2:** Effects of *APOC3* SNPs on serum lipidomics in patients with nonalcoholic fatty liver disease.

Lipids	rs4520			rs4225			rs5128			rs2070666			rs2070667		
	T/T	T/C+C/C	*P*	G/G	G/T+T/T	*P*	C/C	G/G+G/C	*P*	A/A	T/T+T/A	*P*	G/G	G/A+A/A	*P*
Cer 42 : 1; 2	0.238 ± 0.061	0.296 ± 0.088	0.063	0.285 ± 0.088	0.272 ± 0.083	0.676	0.071 ± 0.027	0.065 ± 0.025	0.373	0.258 ± 0.072	0.284 ± 0.087	0.502	0.261 ± 0.079	0.323 ± 0.085	0.049^∗^
DG 36 : 4	0.112 ± 0.095	0.087 ± 0.056	0.362	0.102 ± 0.078	0.086 ± 0.058	0.513	0.081 ± 0.044	0.110 ± 0.089	0.254	0.078 ± 0.030	0.072 ± 0.028	0.630	0.108 ± 0.077	0.061 ± 0.026	0.013^∗^
PCO 38 : 4	0.498 ± 0.075	0.606 ± 0.142	0.007^∗^	0.577 ± 0.162	0.571 ± 0.093	0.899	0.599 ± 0.138	0.546 ± 0.128	0.259	0.535 ± 0.067	0.583 ± 0.144	0.437	0.543 ± 0.112	0.649 ± 0.160	0.034^∗^
PCO 38 : 5	1.057 ± 0.104	1.224 ± 0.239	0.008^∗^	1.164 ± 0.219	1.189 ± 0.230	0.747	0.093 ± 0.026	0.083 ± 0.018	0.074	1.171 ± 0.133	1.176 ± 0.237	0.960	1.162 ± 0.239	1.206 ± 0.175	0.608
PCO 40 : 4	0.072 ± 0.011	0.095 ± 0.023	0.001^∗^	0.089 ± 0.029	0.088 ± 0.013	0.961	0.100 ± 0.032	0.099 ± 0.029	0.227	0.074 ± 0.010	0.091 ± 0.024	0.101	0.083 ± 0.020	0.101 ± 0.025	0.030^∗^
PCO 40 : 5	0.088 ± 0.019	0.104 ± 0.033	0.088	0.104 ± 0.032	0.094 ± 0.028	0.372	0.055 ± 0.017	0.051 ± 0.012	0.897	0.084 ± 0.010	0.103 ± 0.032	0.017^∗^	0.095 ± 0.028	0.110 ± 0.034	0.214
PCO 42 : 4	0.043 ± 0.009	0.057 ± 0.015	0.009^∗^	0.053 ± 0.019	0.052 ± 0.008	0.841	0.118 ± 0.037	0.107 ± 0.030	0.444	0.043 ± 0.008	0.055 ± 0.015	0.089	0.050 ± 0.014	0.060 ± 0.015	0.061
PCO 42 : 5	0.093 ± 0.017	0.120 ± 0.036	0.007^∗^	0.112 ± 0.039	0.113 ± 0.028	0.895	0.133 ± 0.039	0.132 ± 0.041	0.369	0.094 ± 0.015	0.116 ± 0.036	0.025^∗^	0.109 ± 0.032	0.122 ± 0.039	0.320
PCO 44 : 5	0.107 ± 0.024	0.143 ± 0.040	0.003^∗^	0.132 ± 0.045	0.133 ± 0.034	0.900	0.320 ± 0.020	0.315 ± 0.016	0.942	0.105 ± 0.020	0.138 ± 0.040	0.061	0.126 ± 0.036	0.147 ± 0.045	0.176
PE 34 : 0	0.324 ± 0.021	0.315 ± 0.016	0.223	0.321 ± 0.018	0.312 ± 0.018	0.162	0.127 ± 0.055	0.097 ± 0.044	0.467	0.337 ± 0.016	0.313 ± 0.016	0.003^∗^	0.317 ± 0.020	0.317 ± 0.014	0.950
PE 36 : 1	0.113 ± 0.044	0.113 ± 0.055	0.999	0.127 ± 0.060	0.096 ± 0.033	0.064	0.034 ± 0.017	0.029 ± 0.008	0.092	0.154 ± 0.072	0.105 ± 0.043	0.031^∗^	0.110 ± 0.051	0.120 ± 0.055	0.631
PEO 42 : 7	0.032 ± 0.009	0.031 ± 0.015	0.779	0.035 ± 0.015	0.026 ± 0.009	0.046^∗^	0.096 ± 0.040	0.091 ± 0.047	0.294	0.033 ± 0.010	0.031 ± 0.014	0.759	0.032 ± 0.013	0.030 ± 0.015	0.641
PI 36 : 1	0.091 ± 0.027	0.095 ± 0.048	0.794	0.107 ± 0.048	0.077 ± 0.030	0.036^∗^	0.039 ± 0.020	0.033 ± 0.014	0.726	0.106 ± 0.035	0.091 ± 0.044	0.438	0.088 ± 0.036	0.107 ± 0.057	0.249
PI 40 : 5	0.033 ± 0.015	0.037 ± 0.018	0.592	0.042 ± 0.020	0.028 ± 0.008	0.011^∗^	0.090 ± 0.044	0.079 ± 0.026	0.286	0.040 ± 0.025	0.035 ± 0.016	0.662	0.035 ± 0.016	0.039 ± 0.020	0.513
PI 40 : 6	0.083 ± 0.033	0.085 ± 0.039	0.913	0.096 ± 0.043	0.069 ± 0.020	0.023^∗^	0.317 ± 0.229	0.366 ± 0.308	0.394	0.106 ± 0.065	0.080 ± 0.027	0.368	0.084 ± 0.039	0.087 ± 0.032	0.829
TG 54 : 7	0.360 ± 0.279	0.332 ± 0.265	0.781	0.322 ± 0.260	0.364 ± 0.280	0.655	0.030 ± 0.022	0.033 ± 0.029	0.601	0.325 ± 0.316	0.343 ± 0.260	0.882	0.394 ± 0.288	0.210 ± 0.142	0.019^∗^
TG 54 : 8	0.035 ± 0.028	0.030 ± 0.025	0.644	0.029 ± 0.024	0.035 ± 0.028	0.519	0.026 ± 0.008	0.023 ± 0.004	0.728	0.032 ± 0.032	0.031 ± 0.024	0.971	0.036 ± 0.027	0.020 ± 0.015	0.041^∗^
TG 56 : 0	0.026 ± 0.008	0.024 ± 0.006	0.283	0.026 ± 0.006	0.022 ± 0.006	0.039^∗^	0.030 ± 0.017	0.036 ± 0.029	0.296	0.030 ± 0.010	0.023 ± 0.005	0.174	0.024 ± 0.007	0.026 ± 0.005	0.339
TG 56 : 9	0.031 ± 0.020	0.034 ± 0.025	0.746	0.032 ± 0.023	0.033 ± 0.023	0.938	0.071 ± 0.027	0.065 ± 0.025	0.498	0.031 ± 0.022	0.033 ± 0.024	0.800	0.037 ± 0.026	0.023 ± 0.011	0.039^∗^

Cer: ceramide; DG: diacylglycerol; PCO: choline plasmalogen; PE: phosphatidylethanolamine; PEO: ethanolamine plasmalogen; PI: phosphatidylinositol; TG: triacylglycerol. Values are expressed as mean ± SD. ^∗^*P* < 0.05.

## Data Availability

The data used to support the findings of this study are available from the corresponding author upon reasonable request.

## References

[1] Bedossa P. (2017). Pathology of non-alcoholic fatty liver disease. *Liver International*.

[2] Castera L., Friedrich-Rust M., Loomba R. (2019). Noninvasive assessment of liver disease in patients with nonalcoholic fatty liver disease. *Gastroenterology*.

[3] Takahashi Y., Fukusato T. (2014). Histopathology of nonalcoholic fatty liver disease/nonalcoholic steatohepatitis. *World Journal of Gastroenterology*.

[4] Anstee Q. M., Reeves H. L., Kotsiliti E., Govaere O., Heikenwalder M. (2019). From NASH to HCC: current concepts and future challenges. *Nature Reviews. Gastroenterology & Hepatology*.

[5] Byrne C. D., Targher G. (2015). NAFLD: a multisystem disease. *Journal of Hepatology*.

[6] Marengo A., Rosso C., Bugianesi E. (2016). Liver cancer: connections with obesity, fatty liver, and cirrhosis. *Annual Review of Medicine*.

[7] Kolodziejczyk A. A., Zheng D., Shibolet O., Elinav E. (2019). The role of the microbiome in NAFLD and NASH. *EMBO Molecular Medicine*.

[8] Smith A., Baumgartner K., Bositis C. (2019). Cirrhosis: diagnosis and management. *American Family Physician*.

[9] Younossi Z. M., Koenig A. B., Abdelatif D., Fazel Y., Henry L., Wymer M. (2016). Global epidemiology of nonalcoholic fatty liver disease-meta-analytic assessment of prevalence, incidence, and outcomes. *Hepatology*.

[10] Buzzetti E., Pinzani M., Tsochatzis E. A. (2016). The multiple-hit pathogenesis of non-alcoholic fatty liver disease (NAFLD). *Metabolism-Clinical and Experimental*.

[11] Marchesini G., Day C. P., Dufour J. F. (2016). EASL-EASD-EASO Clinical Practice Guidelines for the management of non-alcoholic fatty liver disease. *Journal of Hepatology*.

[12] Musso G., Gambino R., Cassader M. (2009). Recent insights into hepatic lipid metabolism in non-alcoholic fatty liver disease (NAFLD). *Progress in Lipid Research*.

[13] Younossi Z. M., Baranova A., Ziegler K. (2005). A genomic and proteomic study of the spectrum of nonalcoholic fatty liver disease. *Hepatology*.

[14] Malhi H., Gores G. (2008). Molecular mechanisms of lipotoxicity in nonalcoholic fatty liver disease. *Seminars in Liver Disease*.

[15] Kawano Y., Cohen D. E. (2013). Mechanisms of hepatic triglyceride accumulation in non-alcoholic fatty liver disease. *Journal of Gastroenterology*.

[16] Puri P., Baillie R. A., Wiest M. M. (2007). A lipidomic analysis of nonalcoholic fatty liver disease. *Hepatology*.

[17] Hall Z., Bond N. J., Ashmore T. (2017). Lipid zonation and phospholipid remodeling in nonalcoholic fatty liver disease. *Hepatology*.

[18] Tilg H., Moschen A. R. (2010). Evolution of inflammation in nonalcoholic fatty liver disease: the multiple parallel hits hypothesis. *Hepatology*.

[19] Gieger C., Geistlinger L., Altmaier E. (2008). Genetics meets metabolomics: a genome-wide association study of metabolite profiles in human serum. *PLoS Genetics*.

[20] Hallman D. M., Srinivasan S. R., Chen W., Boerwinkle E., Berenson G. S. (2006). Longitudinal analysis of haplotypes and polymorphisms of the APOA5 and *APOC3* genes associated with variation in serum triglyceride levels: the Bogalusa Heart Study. *Metabolism*.

[21] Kanehisa M., Goto S., Hattori M. (2006). From genomics to chemical genomics: new developments in KEGG. *Nucleic Acids Research*.

[22] Kathiresan S., Melander O., Guiducci C. (2008). Six new loci associated with blood low-density lipoprotein cholesterol, high-density lipoprotein cholesterol or triglycerides in humans. *Nature Genetics*.

[23] Li G. P., Wang J. Y., Yan S. K., Chen B. S., Xue H., Wu G. (2004). Genetic effect of two polymorphisms in the apolipoprotein A5 gene and apolipoprotein C3 gene on serum lipids and lipoproteins levels in a Chinese population. *Clinical Genetics*.

[24] Taylor K. C., Carty C. L., Dumitrescu L. (2013). Investigation of gene-by-sex interactions for lipid traits in diverse populations from the population architecture using genomics and epidemiology study. *BMC Genetics*.

[25] Adams L. A., White S. W., Marsh J. A. (2013). Association between liver-specific gene polymorphisms and their expression levels with nonalcoholic fatty liver disease. *Hepatology*.

[26] Chalasani N., Guo X., Loomba R. (2010). Genome-wide association study identifies variants associated with histologic features of nonalcoholic fatty liver disease. *Gastroenterology*.

[27] Kitamoto T., Kitamoto A., Yoneda M. (2013). Genome-wide scan revealed that polymorphisms in the PNPLA3, SAMM50, and PARVB genes are associated with development and progression of nonalcoholic fatty liver disease in Japan. *Human Genetics*.

[28] Feitosa M. F., Wojczynski M. K., North K. E. (2013). The ERLIN1-CHUK-CWF19L1 gene cluster influences liver fat deposition and hepatic inflammation in the NHLBI Family Heart Study. *Atherosclerosis*.

[29] Shang X.-R., Song J.-Y., Liu F.-H., Ma J., Wang H.-J. (2015). GWAS-identified common variants with nonalcoholic fatty liver disease in Chinese children. *Journal of Pediatric Gastroenterology and Nutrition*.

[30] Speliotes E. K., Yerges-Armstrong L. M., Wu J. (2011). Genome-wide association analysis identifies variants associated with nonalcoholic fatty liver disease that have distinct effects on metabolic traits. *Plos Genetics*.

[31] Li M. R., Zhang S. H., Chao K. (2014). Apolipoprotein C3 (-455T>C) polymorphism confers susceptibility to nonalcoholic fatty liver disease in the Southern Han Chinese population. *World Journal of Gastroenterology*.

[32] Petersen K. F., Dufour S., Hariri A. (2010). Apolipoprotein C3 gene variants in nonalcoholic fatty liver disease. *The New England Journal of Medicine*.

[33] Crosby J., Peloso G., Auer P. (2014). Loss-of-function mutations in *APOC3*, triglycerides, and coronary disease. *New England Journal of Medicine*.

[34] Onat A., Hergenc G., Sansoy V. (2003). Apolipoprotein C-III, a strong discriminant of coronary risk in men and a determinant of the metabolic syndrome in both genders. *Atherosclerosis*.

[35] van Dijk K. W., Rensen P. C. N., Voshol P. J., Havekes L. M. (2004). The role and mode of action of apolipoproteins CIII and AV: synergistic actors in triglyceride metabolism?. *Current Opinion in Lipidology*.

[36] Jorgensen A. B., Frikke-Schmidt R., Nordestgaard B. G., Tybjaerg-Hansen A. (2014). Loss-of-function mutations in *APOC3* and risk of ischemic vascular disease. *New England Journal of Medicine*.

[37] Ståhlman M., Fagerberg B., Adiels M. (2013). Dyslipidemia, but not hyperglycemia and insulin resistance, is associated with marked alterations in the HDL lipidome in type 2 diabetic subjects in the DIWA cohort: impact on small HDL particles. *Biochimica et Biophysica Acta*.

[38] Waterworth D. M., Ribalta J., Nicaud V., Dallongeville J., Humphries S. E., Talmud P. (1999). ApoCIII gene variants modulate postprandial response to both glucose and fat tolerance tests. *Circulation*.

[39] Maeda N., Li H., Lee D., Oliver P., Quarfordt S. H., Osada J. (1994). Targeted disruption of the apolipoprotein C-III gene in mice results in hypotriglyceridemia and protection from postprandial hypertriglyceridemia. *Journal of Biological Chemistry*.

[40] Cheng X., Yamauchi J., Lee S. (2017). *APOC3* protein is not a predisposing factor for fat-induced nonalcoholic fatty liver disease in mice. *Journal of Biological Chemistry*.

[41] Kozlitina J., Boerwinkle E., Cohen J. C., Hobbs H. H. (2011). Dissociation between *APOC3* variants, hepatic triglyceride content and insulin resistance. *Hepatology*.

[42] Lee H. Y., Birkenfeld A. L., Jornayvaz F. R. (2011). Apolipoprotein CIII overexpressing mice are predisposed to diet-induced hepatic steatosis and hepatic insulin resistance. *Hepatology*.

[43] Verrijken A., Beckers S., Francque S. (2013). A gene variant of PNPLA3, but not of *APOC3*, is associated with histological parameters of NAFLD in an obese population. *Obesity*.

[44] Vajro P., Lenta S., Socha P. (2012). Diagnosis of nonalcoholic fatty liver disease in children and adolescents: position paper of the ESPGHAN Hepatology Committee. *Journal of Pediatric Gastroenterology and Nutrition*.

[45] Younossi Z. M., Golabi P., de Avila L. (2019). The global epidemiology of NAFLD and NASH in patients with type 2 diabetes: a systematic review and meta-analysis. *Journal of Hepatology*.

[46] Kazankov K., Jorgensen S. M. D., Thomsen K. L. (2019). The role of macrophages in nonalcoholic fatty liver disease and nonalcoholic steatohepatitis. *Nature Reviews Gastroenterology & Hepatology*.

[47] Khan R. S., Bril F., Cusi K., Newsome P. N. (2019). Modulation of insulin resistance in nonalcoholic fatty liver disease. *Hepatology*.

[48] Pan Q., Zhang R. N., Wang Y. Q. (2015). Linked PNPLA3 polymorphisms confer susceptibility to nonalcoholic steatohepatitis and decreased viral load in chronic hepatitis B. *World Journal of Gastroenterology*.

[49] Yang R.-X., Hu C.-X., Sun W.-L. (2017). Serum monounsaturated triacylglycerol predicts steatohepatitis in patients with non-alcoholic fatty liver disease and chronic hepatitis B. *Scientific Reports*.

[50] Bedossa P., the FLIP Pathology Consortium (2014). Utility and appropriateness of the fatty liver inhibition of progression (FLIP) algorithm and steatosis, activity, and fibrosis (SAF) score in the evaluation of biopsies of nonalcoholic fatty liver disease. *Hepatology*.

[51] Bedossa P., Poitou C., Veyrie N. (2012). Histopathological algorithm and scoring system for evaluation of liver lesions in morbidly obese patients. *Hepatology*.

[52] Kleiner D. E., Brunt E. M., van Natta M. (2005). Design and validation of a histological scoring system for nonalcoholic fatty liver disease. *Hepatology*.

[53] Shachter N. S. (2001). Apolipoproteins C-I and C-III as important modulators of lipoprotein metabolism. *Current Opinion in Lipidology*.

[54] Norata G. D., Tsimikas S., Pirillo A., Catapano A. L. (2015). Apolipoprotein C-III: from pathophysiology to pharmacology. *Trends in Pharmacological Sciences*.

[55] Ooi E. M. M., Barrett P. H. R., Chan D. C., Watts G. F. (2008). Apolipoprotein C-III: understanding an emerging cardiovascular risk factor. *Clinical Science*.

[56] Jong M. C., Hofker M. H., Havekes L. M. (1999). Role of ApoCs in lipoprotein metabolism: functional differences between ApoC1, ApoC2, and ApoC3. *Arteriosclerosis Thrombosis and Vascular Biology*.

[57] Lai C. Q., Parnell L. D., Ordovas J. M. (2005). The APOA1/C3/A4/A5 gene cluster, lipid metabolism and cardiovascular disease risk. *Current Opinion in Lipidology*.

[58] Kathiresan S., Willer C. J., Peloso G. M. (2009). Common variants at 30 loci contribute to polygenic dyslipidemia. *Nature Genetics*.

[59] Schunkert H., König I. R., Kathiresan S. (2011). Large-scale association analysis identifies 13 new susceptibility loci for coronary artery disease. *Nature Genetics*.

[60] Webb T. R., Erdmann J., Stirrups K. E. (2017). Systematic evaluation of pleiotropy identifies 6 further loci associated with coronary artery disease. *Journal of the American College of Cardiology*.

[61] Orešič M., Hyötyläinen T., Kotronen A. (2013). Prediction of non-alcoholic fatty-liver disease and liver fat content by serum molecular lipids. *Diabetologia*.

[62] Tanaka N., Matsubara T., Krausz K. W., Patterson A. D., Gonzalez F. J. (2012). Disruption of phospholipid and bile acid homeostasis in mice with nonalcoholic steatohepatitis. *Hepatology*.

[63] Pardina E., Baena-Fustegueras J. A., Catalán R. (2009). Increased expression and activity of hepatic lipase in the liver of morbidly obese adult patients in relation to lipid content. *Obesity Surgery*.

[64] Westerbacka J., Kolak M., Kiviluoto T. (2007). Genes involved in fatty acid partitioning and binding, lipolysis, monocyte/macrophage recruitment, and inflammation are overexpressed in the human fatty liver of insulin-resistant subjects. *Diabetes*.

[65] Silva Figueiredo P., Carla Inada A., Marcelino G. (2017). Fatty acids consumption: the role metabolic aspects involved in obesity and its associated disorders. *Nutrients*.

[66] Yamada K., Mizukoshi E., Sunagozaka H. (2015). Response to importance of confounding factors in assessing fatty acid compositions in patients with non-alcoholic steatohepatitis. *Liver International*.

[67] Ajuwon K. M., Spurlock M. E. (2005). Palmitate activates the NF-kappaB transcription factor and induces IL-6 and TNFalpha expression in 3T3-L1 adipocytes. *The Journal of Nutrition*.

[68] Tanaka N., Zhang X., Sugiyama E. (2010). Eicosapentaenoic acid improves hepatic steatosis independent of PPAR*α* activation through inhibition of SREBP-1 maturation in mice. *Biochemical Pharmacology*.

[69] Liu L., Hu Q., Wu H. (2018). Dietary DHA/EPA ratio changes fatty acid composition and attenuates diet-induced accumulation of lipid in the liver of ApoE(-/-) mice. *Oxidative Medicine and Cellular Longevity*.

[70] Dossi C. G., Tapia G. S., Espinosa A., Videla L. A., D'Espessailles A. (2014). Reversal of high-fat diet-induced hepatic steatosis by n-3 LCPUFA: role of PPAR-*α* and SREBP-1c. *The Journal of Nutritional Biochemistry*.

[71] Hyysalo J., Stojkovic I., Kotronen A. (2012). Genetic variation in PNPLA3 but not *APOC3* influences liver fat in non-alcoholic fatty liver disease. *Journal of Gastroenterology and Hepatology*.

[72] Imgenberg-Kreuz J., Carlsson Almlöf J., Leonard D. (2018). DNA methylation mapping identifies gene regulatory effects in patients with systemic lupus erythematosus. *Annals of the Rheumatic Diseases*.

[73] Bradley R. G., Binder E. B., Epstein M. P. (2008). Influence of child abuse on adult depression: moderation by the corticotropin-releasing hormone receptor gene. *Archives of General Psychiatry*.

[74] Shieh M., Chitnis N., Clark P., Johnson F. B., Kamoun M., Monos D. (2019). Computational assessment of miRNA binding to low and high expression HLA-DPB1 allelic sequences. *Human Immunology*.

[75] Hecker M., Boxberger N., Illner N. (2019). A genetic variant associated with multiple sclerosis inversely affects the expression of CD58 and microRNA-548ac from the same gene. *PLoS Genetics*.

[76] Euskirchen G., Royce T. E., Bertone P. (2004). CREB binds to multiple loci on human chromosome 22. *Molecular and Cellular Biology*.

